# Assessment of Vascular Circulation in Alopecia Areata Using the FMSF Technique

**DOI:** 10.3390/jcm14103469

**Published:** 2025-05-15

**Authors:** Anna Woźniacka, Kamila Tokarska, Bartlomiej Żmuda

**Affiliations:** Department of Dermatology and Venereology, Medical University of Lodz, 90-419 Łódź, Poland; kamka1990@o2.pl (K.T.); zmudabartek98@gmail.com (B.Ż.)

**Keywords:** alopecia areata, pathogenesis of alopecia areata, FMSF

## Abstract

**Background:** Alopecia areata is regarded as a T cell-mediated autoimmune disorder, but the exact etiopathogenesis of the disease has not been completely elucidated. The aim of the study was to assess vascular circulation using Flow-Mediated Skin Fluorescence (FMSF) in alopecia patients compared to healthy volunteers, which could explain disease pathogenesis. **Methods:** FMSF is a new non-invasive method for assessing vascular circulation. The study recruited thirty women and four men. In our group, the most common clinical pattern of hair loss was alopecia with circular patches (AA), recognizable in 26 patients: twenty-two women and four men. Alopecia universalis (AU) was diagnosed in eight patients: all women. **Results:** The most pronounced differences between experimental group participants and controls are seen in the flowmotion (FM), neurogenic oscillation (NEURO), and normoxia oscillatory index (NOI) parameters characterizing microcirculation oscillations. In alopecia, microcirculation oscillations characterized by the FM and NEURO parameters are significantly decreased. **Conclusions:** This observation may suggest that neuroinflammation is an important factor responsible for alopecia pathogenesis. The women with alopecia areata have dysfunctional microcirculatory function. FMSF could serve as a useful tool for monitoring patients with alopecia.

## 1. Introduction

Alopecia areata is considered a T cell-mediated autoimmune condition that causes non-scarring hair loss, mainly on the scalp but possibly affecting other hair-bearing areas of the body as well. The disease, which affects approximately 2% of the global population, can substantially decrease a patient’s quality of life [1]. Although the exact etiopathogenesis of the disease has not been completely elucidated, it is currently believed that genetic, environmental, immune, vascular, and mental factors may play a role in the development of the disease. Patients with alopecia areata have a higher risk of different comorbidities and emotional stress that can lead to decreased blood flow and could be regarded as a potential trigger factor. Prolonged psychological stress is recognized as a significant risk factor affecting the entire vascular system. Trauma and anxiety are linked to elevated circulating levels of norepinephrine, which can induce microvascular vasoconstriction [2]. Elevated catecholamine levels and reduced blood flow may contribute to the upregulation of corticotropin-releasing hormone receptors in the skin surrounding hair follicles [3].

Skin is strategically positioned at the interface with the external environment. Beyond serving as a physical barrier, it actively detects, integrates, and responds to a wide range of stressors, including biological agents, ultraviolet radiation, and various physical and chemical stimuli. It is increasingly recognized as a key peripheral neuro-endocrine-immune organ, intricately connected to central regulatory systems. Furthermore, both epidermal and dermal cells are capable of producing and responding to classical stress-related neurotransmitters, neuropeptides, and hormones.

Flow-Mediated Skin Fluorescence (FMSF) represents an advanced, non-invasive sensory system for evaluating vascular circulation. This technique quantifies the dynamic fluctuations in NADH fluorescence emission from skin tissue, providing critical data on mitochondrial metabolic activity and the efficiency of intracellular oxygen delivery via the circulatory system. Vascular assessment using FMSF is based on a few parameters, including the reactive hyperemia response (RHR), hypoxia sensitivity (HS), and the normoxia oscillatory index (NOI). As the NOI is particularly instrumental in evaluating microcirculatory dynamics under conditions of stress induced by various factors, including emotional strain, physical exertion, or post-infectious recovery, this parameter was within a special spectrum of our interest [4].

The aim of the study was to assess clinical parameters, co-morbidities, and FMSF parameters in AA patients in comparison to healthy volunteers to explain the complex disease pathogenesis.

## 2. Materials and Methods

We conducted a case–control study at the Department of Dermatology and Venereology at the Medical University of Lodz, Poland. All participants signed a written informed consent form before the procedures started. The diagnosis of AA was performed by a board-certified dermatologist (AW). The study recruited 30 women and 4 men, aged from 18 to 77 years. In our study group, the most common clinical pattern of hair loss was alopecia areata with circular patches located on the scalp or beard (AA). The study group consisted of 26 patients: 22 women and 4 men. Alopecia universalis (AU) is generally regarded as the most severe form of alopecia areata. It comprises the loss of all body hair, including eyebrows, eyelashes, chest hair, armpit hair, and pubic hair (8 patients; all women).

The healthy control group (female; *n* = 17; mean age 44.6) was randomly selected from patients attending the Dermatology Department with non-inflammatory dermatological conditions, hospital staff, and their relatives. Inclusion criteria required participants to be 18 years or older and free from a personal or family history of alopecia areata or any autoimmune disorders.

All participants underwent a thorough medical examination. The following data were collected: sex, age, BMI, smoking habits, diagnosis of comorbidities (cardiovascular diseases, dyslipidemia, diabetes, and metabolic syndrome), and drug intake (Table 1). All blood samples were drawn after at least 8 h of fasting. Blood parameters were measured using standard automated techniques for evaluation: complete blood count, C-reactive protein, glucose level, IL-6, high-density lipoprotein, triglycerides, total cholesterol, low-density lipoprotein cholesterol, and liver enzymes.

### 2.1. FMSF Methodology

Flow-Mediated Skin Fluorescence (FMSF) measurements were performed using the AngioExpert system (Angionica Ltd., Lodz, Poland), a novel, non-invasive diagnostic tool designed for the assessment of vascular function and metabolic regulation. This method quantifies dynamic changes in the fluorescence of reduced nicotinamide adenine dinucleotide (NADH) emitted from the skin, providing real-time data on mitochondrial metabolic status and the efficiency of intracellular oxygen delivery through the circulatory system. NADH, along with its oxidized form NAD⁺, plays a pivotal role in cellular redox reactions and energy metabolism, making it a critical biomarker in physiological and pathophysiological studies.

**Figure 1:**
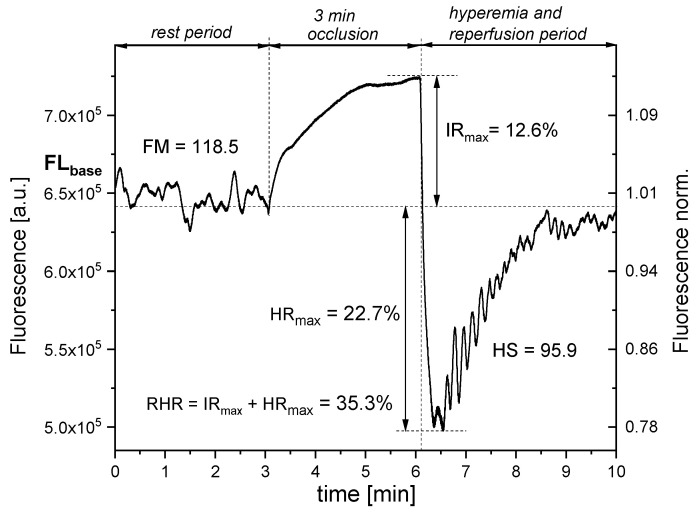
An example FMSF trace recorded for the healthy volunteer (female; 41 y.; no comorbidities).

Before the test, the patient’s blood pressure was measured; then, the patient’s forearm was positioned under the measuring window. The baseline fluorescence was recorded for 3 min prior to the inflation of the occlusion cuff, which was inflated to a pressure of 60 mmHg above the subject’s systolic blood pressure. This induced ischemia, leading to a subsequent increase in NADH fluorescence. After 3 min, the cuff was automatically deflated, causing a rapid decline in NADH fluorescence below baseline levels, followed by a gradual recovery to the resting value. This post-occlusion phase, termed the hyperemic response, was divided into two distinct phases. The initial phase, lasting approximately 20–30 s, was associated with reactive hyperemia and a rapid reduction in NADH fluorescence. This was followed by the reperfusion phase, during which NADH fluorescence progressively returned to baseline levels. Both the baseline and reperfusion phases exhibited characteristic microcirculatory oscillations, as depicted in Figure 1.

Prior to occlusion and during the 3 min period following post-occlusive reactive hyperemia (PORH), the FMSF method enables the detection of oscillatory patterns in microcirculatory blood flow, commonly referred to as flowmotion (FM). This allows for the assessment of microcirculatory oscillatory responses to transient hypoxia, which is critical for understanding microvascular function in pathologies associated with ischemia and oxygen deficiency. The amplitude and frequency of these oscillations, embedded within the FMSF signal, can be quantified using the Fast Fourier Transform (FFT) algorithm.

The resulting frequency components are categorized into three distinct groups that reflect the physiological activity of specific regulatory mechanisms: endothelial (<0.021 Hz), neurogenic (0.021–0.052 Hz), and myogenic (0.052–0.15 Hz) origins [4]. In the manuscript, other parameters were also evaluated.

The normoxia oscillatory index (NOI) characterizes microcirculatory function by quantifying the proportion of endothelial and neurogenic oscillatory components under baseline, normoxic conditions. These low-frequency oscillations reflect the intrinsic rhythmic activity of vascular tone regulation, with the endothelial component associated with nitric oxide (NO)-mediated vasodilation and the neurogenic component linked to sympathetic nervous system activity. A reduced NOI is indicative of increased peripheral vasoconstriction and impaired endothelial or autonomic function, and it serves as a sensitive marker of fatigue-related vascular dysregulation caused by emotional stress, physical exertion, or post-infectious conditions.

The reactive hyperemia response (RHR) is a functional parameter used to assess macrovascular endothelial function by quantifying the vasodilatory response of large and medium-sized arteries following a period of transient ischemia. A reduced RHR indicates impaired NO-mediated vasodilation and is indicative of endothelial dysfunction, a key pathophysiological feature in a range of cardiovascular and metabolic diseases, including atherosclerosis, hypertension, and diabetes mellitus.

The IR (ischemic response) characterizes the peak increase in NADH fluorescence during the occlusion phase and serves as an indicator of the tissue’s response to ischemia. This parameter reflects mitochondrial redox status and provides a direct measure of mitochondrial efficiency or dysfunction under hypoxic conditions.

The HR (hyperemic response) corresponds to the maximum decrease in NADH fluorescence following the release of occlusion in the brachial artery. It reflects the rapid restoration of oxygen supply and mitochondrial reoxidation, serving as a marker of vascular reactivity and metabolic recovery during post-ischemic reperfusion.

Hypoxia sensitivity (HS) represents the proportion of the FM(R) signal—flowmotion activity recorded during the reperfusion phase—attributable to myogenic oscillations. The HS parameter thus offers a quantitative assessment of the microcirculatory response to hypoxia and serves as a sensitive marker of myogenic regulatory function within the microvasculature [5].

### 2.2. Statistical Analysis

Data acquisition and preliminary analysis were conducted using proprietary software integrated into the AngioExpert system. Subsequent statistical analyses were carried out using OriginPro 2023 (OriginLab Corporation, Northampton, MA, USA). The Shapiro–Wilk test was employed to assess the normality of data distribution. Depending on the distribution characteristics, group comparisons were performed using either the two-tailed Student’s *t*-test or the Mann–Whitney U test. Correlation analyses were conducted using Pearson’s or Spearman’s correlation coefficients, as appropriate. A *p*-value of less than 0.05 was considered statistically significant.

## 3. Results

### 3.1. Modification of the Analyzed Target Group

The analysis of clinical data, especially co-morbidity and biochemical parameters, showed significant heterogeneity of the analyzed group. Therefore, in order to exclude factors that could have a non-specific effect on the analyzed FMSF parameters, a few patients, 60.0 ± 14.3 years of age, with serious comorbidities were excluded from further analyses.

In addition, data from the literature indicate that some differences in the analyzed parameters may also be gender-dependent [6]. Therefore, due to the small number of men and the lack of possibility to carry out a separate statistical assessment, the group of men was ultimately not taken into account either.

Initially, for the study, a group of women with alopecia areata was recruited, comprising twenty women with AA and eight with AU. Among this group, six elderly women (four with AA and two with AU, aged above 60.0 years) were identified with evident mitochondrial dysfunction characterized by a negative ischemic response (IRmax < 0). A careful examination of the clinical characteristics of this subgroup suggested that alopecia areata is seriously masked by the observed mitochondrial dysfunction, so this subgroup was excluded from further analysis [6]. Finally, twenty-four women with alopecia areata (eighteen with AA and six with AU) were considered for the assessment of vascular circulation and metabolic regulation. Characteristics of the studied group are presented in Table 1. This group, based on an analysis of the IRmax parameter, is essentially free from noticeable mitochondrial dysfunction (IRmax > 0) and is similar to the control group in that aspect (Table 2). Such conditions should allow the distinct discrimination of vascular effects between the alopecia areata and control groups.

### 3.2. FMSF Results

**Figure 2:**
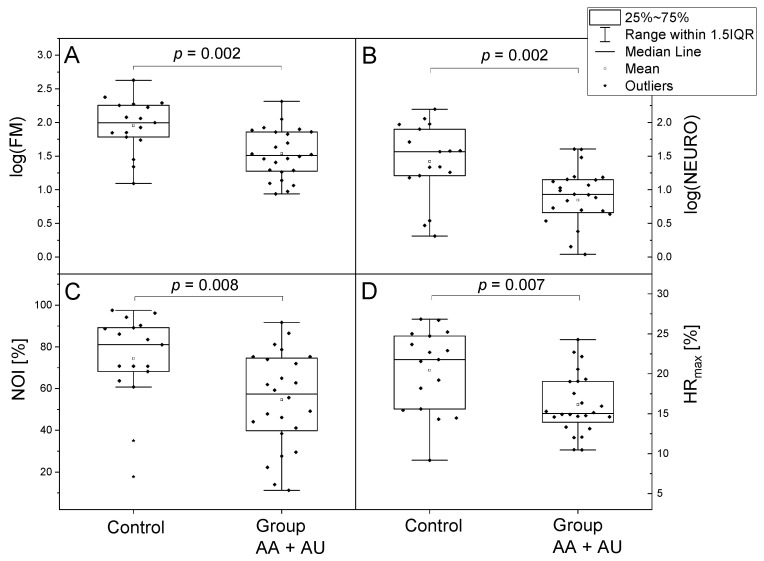
A comparison of the log(FM) (**A**), log(NEURO) (**B**), NOI (**C**), and HR_max_ (**D**) parameters for a group of alopecia patients (female; *n* = 24; mean age 47.9 (17–75 y.)), and a control group (female; *n* = 17; mean age 44.6 (30–50 y.)). The differences between the parameters of the compared groups were considered statistically significant when *p* < 0.05. The *p*-values were calculated from a two-sample *t*-test for comparisons (**A**,**B**,**D**) and the Mann–Whitney test for comparison (**C**).

A careful comparison of the FMSF parameters presented in Table 2 and Figure 2 revealed statistically significant differences in vascular circulation between the alopecia areata and control groups. The most pronounced effects are seen in the FM, NEURO, and NOI parameters, characterizing microcirculation oscillations. Clearly, in both the AA and AU subgroups, the microcirculation oscillations characterized by the FM and NEURO parameters are seriously decreased.

FM is a parameter used to assess microcirculation. The results of the study indicate that this parameter is statistically significantly lower in patients with AA compared to the control group (*p* = 0.002). Moreover, the values of this parameter were almost twice as low in the AU group (27.1 ± 25.9) compared to the AA group (55.2 ± 47.3).

A particularly serious drop is seen in the NEURO parameter (*p* = 0.002), characterizing neurogenic microcirculatory oscillations. It is worth emphasizing that the NEURO parameter is almost twice as low in patients with AU (6.3 ± 6.4) compared to patients with AA (13.2 ± 11.7).

Significant statistical differences between the experimental group and control group are observed in the NOI parameter (*p* = 0.008). Unlike the previous parameters, the difference between the AA and AU group is not significant.

The HRmax parameter, used for the assessment of macrocirculatory dysfunction and prediction of the risk of cardiovascular diseases development, is decreased in alopecia areata patients in comparison to healthy volunteers (*p* = 0.0.007). However, no statistically significant differences are noted between the two patient subpopulations.

In terms of the other parameters assessed (RHR, IRmax, and HS), no statistically significant differences are observed, either between women with alopecia and the healthy volunteers or between the two subgroups of patients.

## 4. Discussion

Alopecia is a multifactorial disease with a complex and incompletely understood etiology, in the course of which genetically predisposed people experience hair loss. Literature data indicate that the disease also has an inflammatory and autoimmune basis, in which Th1, Th2, and Th17 cytokines play an important role. Different theories have been proposed. Some researchers claim that local dysfunction could be a part of systemic inflammatory changes. This theory can be confirmed by the increased serum concentrations of IL-6, IL-8, and IFN gamma. This disorder is visible, especially in people with a more severe process and longer course of the disease [7]. In our study, such correlations were not observed.

Additional studies suggest that the process may be predominantly localized and mediated by neurogenic inflammation, which arises from the activation of peripheral nerve endings and the release of neuropeptides that arrange cutaneous inflammatory responses.

This mechanism involves complex interactions with various cell types, including keratinocytes, Langerhans cells, endothelial cells, and mast cells. Significant differences in the number of neuropeptides in alopecia patients compared to healthy people were shown. This applies to substance P (SP), neuropeptide Y, vasoactive intestinal peptide (VIP), melanocyte-stimulating hormone (MSH), corticotropin-releasing hormone (CRH), somatostatin, and calcitonin gene-related peptide (CGRP). Afferent fibers, autonomic nerve fibers, myelin type Aδ fibers, and unmyelinated C-fibers are densely distributed in all layers of the skin, forming part of the skin’s neuroendocrine system and releasing neuropeptides according to various factors. Neuropeptides—including neuromodulators, neurotransmitters, and neurohormones—play a critical role in modulating immune responses by changing neurological impulses from afferent nerve fibers into biochemical signals recognizable by immunocompetent cells, such as lymphocytes and mast cells. This neuroimmune communication has the potential to amplify the inflammatory response [8].

The FMSF technique is useful for assessing circulatory status by evaluating parameters reflecting the degree of ischemia and reperfusion of skin cells dependent on blood flow in the vessels. Observation of microcirculation oscillations, especially changes in amplitude and frequency, may signal abnormalities in microcirculation, which are present in the course of many different diseases in terms of their etiopathogenesis. Therefore, we had to exclude patients with serious cardiovascular diseases, in the course of which deviations in the assessed parameters depended on serious heart disease and overlapped/masked deviations in the parameters in the course of alopecia.

The FMSF method permits the recognition of disorders at an early stage of disease development and allows monitoring of the treatment process. In the case of alopecia, it elucidates the elements of the pathogenesis of the disease. Perhaps, in the future, it will be useful for forecasting the course of the disease. It is well known that there are patients who do not respond to treatment, or the disease may disappear spontaneously.

There is a widely accepted theory that emotional stress can lead to increased catecholamines and decreased blood flow, and it has been associated with the upregulation of corticotropin-releasing hormone receptors in the skin around hair follicles [3]. Therefore, in this study, we tried to examine microvascular flow perturbation, using a new AngioExpert device, as a reliable diagnostic method for alopecia pathogenesis assessment.

Microcirculation oscillations (FM) are an important functional element of the circulatory system that allows blood to flow efficiently through small blood vessels. The results of our study indicate that microcirculation disorders can also contribute to the development of alopecia.

Until now, microcirculation disorders have been assessed using Laser Doppler Flowmetry (LDF) and Laser Speckle Contrast Imaging (LSCI). Human microcirculation is commonly studied at the level of the skin, not only due to its accessibility but also because cutaneous microcirculatory dysfunction reflects impairments in systemic microcirculation and blood flow oscillations. A study published by Marcinek et al. pointed out that it is also possible to test microvascular blood circulation via changes in skin biochemistry, especially the mitochondrial NADH redox state of the dermal and/or epidermal cells, which depends on blood circulation. Based on this effect, the FMSF method was developed [4].

FMSF has its limitations, of course. It is a method with high sensitivity and low specificity; therefore, it can be used as a screening test that distinguishes people with circulatory disorders. This is not a method used to diagnose specific diseases; moreover, it is not useful for diagnosing specific skin diseases, but it may prove useful in explaining their pathogenesis. FMSF is not yet included in everyday clinical practice; therefore, there are no established and generally accepted standards, and the results contained in publications refer to a comparative group of healthy people.

Data from the literature indicate that impaired microvascular flow can be observed in various systemic diseases, including cancer, diabetes, neurodegenerative, and cardiovascular illnesses [9,10]. The body’s desire to maintain homeostasis and ensure constant blood flow means that these mechanisms are regulated on many levels.

Oscillations are defined as repetitive or periodic fluctuations, typically occurring over time and having a measurable parameter around a central value. The FM parameter is based on the assessment of oscillations in terms of the mean square deviations of the experimental signal (at sampling frequency of 25 Hz) from the baseline. While the FM parameter exhibits considerable variability, its logarithmic transformation [log(FM)] follows a normal distribution. The results demonstrated a statistically significant reduction in the intensity of low-frequency microcirculatory oscillations, as indicated by both the FM parameter and log(FM), confirming disturbances in cutaneous blood flow.

A careful comparison of the FMSF parameters presented in Table 2 and Figure 2 allows the formulation of some conclusions concerning the observed differences in vascular circulation between the alopecia areata and control groups. The most pronounced effects are seen in the FM, NEURO, and NOI parameters, characterizing microcirculation oscillations, which may indicate their active participation in the pathogenesis of the disease. Clearly, in both the AA and AU subgroups, the microcirculation oscillations characterized by the FM and NEURO parameters are seriously decreased, as seen in Table 2 and Figure 2A,B.

Neurogenic inflammation resulting from neuroimmune dysregulation in the skin may disrupt the inflammatory microenvironment of the hair follicle, a critical factor in the pathogenesis of alopecia areata. Current evidence suggests that mast cell degranulation initiates neurogenic inflammation through the release of various signaling molecules, thereby impairing hair growth and accelerating the transition of hair follicles through their growth cycle. This form of inflammation is triggered by nerve activation, leading to the release of neuropeptides and subsequently causing rapid plasma extravasation and tissue edema [11].

Interestingly, a particularly serious drop is seen in the NEURO parameter characterizing neurogenic microcirculatory oscillations. This observation clearly suggests participation of neuroinflammation in the pathogenesis of the alopecia areata. More pronounced changes in the NEURO and FM parameters in the AU subgroup compared to the AA subgroup indicate that the microvascular dysfunction is much more affected in the AU subgroup. The less than two-fold difference between the values of these parameters in AA/AU patients indicates that they may not only be important in the pathogenesis of the disease but may also be useful in assessing its activity and prognosis, i.e., monitoring patients during therapy.

The contribution of neurogenic inflammation has added a new dimension to our understanding of the pathogenesis of various chronic cutaneous diseases, including psoriasis, atopic dermatitis, and sensitive skin [11,12,13]. It is generally accepted that stress can trigger and worsen many inflammatory skin diseases, including psoriasis, atopic dermatitis, and alopecia. Stress can affect skin function directly or through the peripheral nervous system, endocrine system, and immune system [14].

The NOI parameter can serve as a good marker for characterizing stress of various origins. It reflects the contribution of endothelial and neurogenic oscillations relative to all oscillations detected within low-frequency intervals (<0.15 Hz). In contrast to FM, the NOI parameter is not age-dependent. A previously performed study revealed that in people with emotional or post-infection stress, the NOI parameter had a value below 60% compared to the healthy population [4].

In alopecia areata, significant statistical differences between the study and control groups were observed in the NOI parameter (*p* = 0.008), but the difference between the AA and AU groups was not significant. This may mean that this parameter reflects individual susceptibility to stress and can act as a trigger factor but does not necessarily correlate with the activity or intensity of the disease process. Therefore, monitoring its activity and course seems to be of little use.

Following occlusion, the cuff pressure is released, and NADH fluorescence decreases below baseline, reaching a minimum before returning to baseline levels (Figure 1). This phase, known as the hyperemic response (HR), is characterized by a rapid decline in NADH fluorescence due to hyperemia. The key parameter defining this phase is HRmax, which represents the maximum decrease in NADH fluorescence. The diagnostic significance of the HRmax parameter is linked to dysfunction in microcirculation. It is usually connected with the increased risk of developing cardiovascular diseases, which could coexist with AA as a comorbid condition. Although alopecia areata is usually considered an organ-specific disease limited to the hair follicle, recent data have highlighted its systemic nature. Some studies have shown that inflammatory dysregulation in patients with immune-mediated conditions is associated with an increased risk of cardiovascular disease. Chronic, systemic inflammation may act as an additional risk factor, contributing to the development of accelerated atherosclerosis in the arterial wall. Endothelial dysfunction and hemodynamic disturbances in regions of turbulent blood flow promote the expression of adhesion molecules and local inflammatory cytokines, such as IL-6 and IL-8. Patricia Burgos-Blasco and her co-workers revealed that AA patients had an increased prevalence of subclinical atherosclerosis compared to healthy controls [15]. Our obtained results, showing statistically significantly lower HRmax values, seem to confirm this hypothesis.

The HS parameter, representing microcirculatory oscillations in response to hypoxia, is mainly related to myogenic oscillations. It is recorded during the reperfusion phase and is responsible for the increased activity of the vessels after post-occlusive reactive hyperemia. While the HS parameter exhibits considerable variability, its logarithmic transformation [log(HS)] follows a normal distribution. Lower values might indicate some microvascular complications, the influence of the myogenic response, and a weak response to ischemia in alopecia patients.

The literature suggests that patients with a negative ischemic response (IR < 0) could be readily recognizable and should receive special medical attention. Previous research has indicated that in females, deviations in the observed FMSF parameters may also be associated with hormonal imbalances [16,17]. Due to the small size of the male group and possible dependence of the analyzed parameters on gender, statistical analyses were carried out only in the group of women. In the future, the study will be expanded. In our selected female study group, there were no statistically significant differences between the patients and the control group, which seems to indicate the lack of significance of HS on the development of alopecia.

The analysis of the IRmax and HS parameters listed in Table 2 suggests quite similar metabolic regulation in the alopecia areata and control groups. However, a few cases among elderly women with evident mitochondrial dysfunction (IRmax < 0) were identified, and such initial observation deserves further evaluation. It is well known that alopecia areata is associated with co-morbidity. Hence, many patient conditions were accompanied by other autoimmune diseases. We excluded only those patients whose IRmax was less than zero, which was associated with serious mitochondrial dysfunction and was the result of overlapping changes in the course of severe systemic disease.

There are some noticeable differences when comparing the HRmax parameter characterizing macrocirculation for the alopecia areata vs. control groups (Table 2 and Figure 2D). However, such changes are much less pronounced compared to the changes seen for microcirculation. In addition, HRmax parameters for the AA and AU groups are quite similar. The results of this study, completed on patients with alopecia areata, are pioneering. Therefore, it is sometimes difficult to determine all relationships in the first observation.

## 5. Conclusions

Interpreting the parameters and dynamics of the NADH fluorescence signal emitted from skin cells in response to occlusion, ischemia, and subsequent hyperemia enables the identification of vascular disturbances that can lead to the development of various diseases, including alopecia areata. In this study, the assessment of vascular circulation, by means of FM, neurogenic oscillations, the NOI, and HRmax, may explain the vascular and neurogenic origin of the autoimmune process.

Women with alopecia areata have seriously dysfunctional microcirculatory function. A particularly pronounced effect is seen in the decreased values of the NEURO parameter, characterizing the intensity of neurogenic microcirculatory oscillations. This observation may suggest that neuroinflammation is an important factor responsible for alopecia areata pathogenesis. A more pronounced decrease in the NEURO parameter was seen in the AU group compared to the AA group, suggesting similar disease etiology abnormalities but enhanced severity. Macrovascular circulation characterized by the HRmax parameter is less affected in the alopecia areata group compared to the control group; however, such an outcome is much weaker compared to that seen in microvascular function.

A significant reduction in the values of the above blood flow parameters helps us to know more about the complex origin of the disease. The analysis of the components of the oscillation and their interrelationships offers some new answers and may shed light on the incompletely understood issues related to the etiopathogenesis of alopecia.

Research on the elucidation of the causes and mechanisms of the disease is of fundamental importance in the design of new therapeutic methods and introduction of personalized therapy. Perhaps in the future, FMSF will be useful for monitoring patients and forecasting the course of the disease.

## Figures and Tables

**Table 1:** Characteristics of the studied groups.

	Group AA	Group AU	Control
N	18	6	17
Age [years]	47.0 ± 16.9	50.7 ± 23.4	44.6 ± 12.0
BMI [kg/m^2^]	25.6 ± 4.7	25.3 ± 3.9	23.9 ± 3.2
DBP [mm Hg]	81.5 ± 8.9	77.5 ± 11.6	76.5 ± 7.4
SBP [mm Hg]	124.8 ± 16.2	129.7 ± 19.4	117.9 ± 11.6
Smoking	2 (11.1)	2 (33.3)	1 (5.9)
Depression	3 (16.7)	2 (33.3)	-
Hashimoto	5 (27.8)	2 (33.3)	-
Hypertension	3 (16.7)	2 (33.3)	-
Hypercholesterolemia	0 (0.0)	1 (16.7)	-
Diabetes	0 (0.0)	1 (16.7)	-

Note: continuous variables—mean ± SD; dichotomous variables—*n* (%). Abbreviations: BMI—body mass index; DBP—diastolic blood pressure; SBP—systolic blood pressure.

**Table 2:** Measured FMSF parameters for the studied groups.

	Group AA	Group AU	Group AA + AU	Control
FM	55.2 ± 47.3	27.1 ± 25.9	48.2 ± 44.2	125.2 ± 101.8
NEURO	13.2 ± 11.7	6.3 ± 6.4	11.4 ± 10.9	47.3 ± 45.0
NOI [%]	55.5 ± 23.3	53.3 ± 24.1	54.6 ± 22.9	74.4 ± 21.6
RHR [%]	27.8 ± 7.7	31.0 ± 8.9	28.6 ± 7.9	33.1 ± 7.9
IR_max_ [%]	11.6 ± 6.1	15.3 ± 7.8	12.5 ± 6.6	13.8 ± 5.6
HR_max_ [%]	16.3 ± 4.1	15.8 ± 2.8	16.1 ± 3.7	20.4 ± 5.1
HS	62.3 ± 59.4	67.6 ± 74.8	63.6 ± 61.9	84.3 ± 78.0

Note: continuous variables—mean ± SD; dichotomous variables—*n* (%). Abbreviations: FM—flowmotion at baseline; NEURO—neurogenic microcirculatory oscillations at baseline; NOI—normoxia oscillatory index; RHR—reactive hyperemia response; IR_max_—ischemic response; HR_max_—hyperemic response; HS—hypoxia sensitivity.

## Data Availability

The data that support the findings of this manuscript are available from the following repositories: SCOPUS (https://www.scopus.com, accessed on 1 March 2025) and PUBMED (https://pubmed.ncbi.nlm.nih.gov/, accessed on 1 March 2025). All relevant articles included in this manuscript can be accessed through these databases.

## References

[1] Teruki D., Masashi I., Yo K. (2023). Alopecia areata: What’s new in the epidemiology, comorbidities, and pathogenesis?. J. Dermatol. Sci..

[2] Jaskanwal Deep Singh S., Takumi T., Ali A., Clark M.M., Wesley P.G., Lerman L.O., Lerman A. (2022). Mental stress and its effects on vascular health. Mayo Clin. Proc..

[3] Katsarou-Katsari A., Singh L.K., Theoharides T.C. (2001). Alopecia areata and affected skin CRH receptor upregulation induced by acute emotional stress. Dermatology.

[4] Marcinek A., Katarzyńska J., Sieron L., Skokowski R., Zieliński J., Gębicki J. (2023). Non-invasive assessment of vascular circulation based on flow mediated skin fluorescence (FMSF). Biology.

[5] Marcinek A., Katarzynska J., Cypryk K., Los-Stegienta A., Slowikowska-Hilczer J., Walczak-Jedrzejowska R., Zielinski J., Gebicki J. (2024). Assessment of Microvascular Function Based on Flowmotion Monitored by the Flow-Mediated Skin Fluorescence Technique. Biosensors.

[6] Marcinek A., Katarzyńska J., Gębicki J. (2025). Simultaneous assessment of mitochondrial and vascular function using the Flow Mediated Skin Fluorescence technique. Front. Physiol..

[7] Glickman J.W., Dubin C., Renert-Yuval Y., Dahabreh D., Kimmel G.W., Auyeung K., Estrada Y.D., Singer G., Krueger J.G., Pavel A.B. (2021). Cross-sectional study of blood biomarkers of patients with moderate to severe alopecia areata reveals systemic immune and cardio-vascular biomarker dysregulation. J. Am. Acad. Dermatol..

[8] Marek-Jozefowicz L., Nedoszytko B., Grochocka M., Żmijewski M.A., Czajkowski R., Cubała W.J., Slominski A.T. (2023). Molecular Mechanisms of Neurogenic Inflammation of the Skin. Int. J. Mol. Sci..

[9] Katarzynska J., Borkowska A., Los A., Marcinek A., Cypryk K., Gebicki J. (2020). Flow-Mediated Skin Fluorescence (FMSF) Technique for Studying Vascular Complications in Type 2 Diabetes. J. Diabetes Sci. Technol..

[10] Katarzynska J., Cholewinski T., Sieron L., Marcinek A., Gebicki J. (2020). Flowmotion Monitored by Flow Mediated Skin Fluorescence (FMSF): A Tool for Characterization of Microcirculatory Status. Front. Physiol..

[11] Shi Y., Wan S., Song X. (2024). Role of neurogenic inflammation in the pathogenesis of alopecia areata. J. Dermatol..

[12] Costa A., Eberlin S., Polettini A.J., Pereira A.F.d.C., Pereira C.S., Ferreira N.M.C., Dolis E., Torloni L.B.O. (2014). Neuromodulatory and Anti-Inflammatory Ingredient for Sensitive Skin: In Vitro Assessment. Inflamm. Allergy Drug Targets.

[13] Holmes A.D., Steinhoff M. (2017). Integrative Concepts of Rosacea Pathophysiology, Clinical Presentation and New Therapeutics. Exp. Dermatol..

[14] Slominski A.T., Slominski R.M., Raman C., Chen J.Y., Athar M., Elmets C. (2022). Neuroendocrine signalling in the skin with a special focus on the epidermal neuropeptides. Am. J. Physiol. Cell Physiol..

[15] Burgos-Blasco P., Gonzalez-Cantero A., Hermosa-Gelbard A., Jiménez-Cahue J., Buendía-Castaño D., Berna-Rico E., Abbad-Jaime de Aragón C., Vañó-Galván S., Saceda-Corralo D. (2024). Subclinical Atherosclerosis in Alopecia Areata: Use-fulness of Arterial Ultrasound for Disease Diagnosis and Analysis of Its Relationship with Cardiometabolic Parameters. J. Clin. Med..

[16] Marcinek A., Katarzynska J., Gebicki J. (2024). A New Approach to Vascular Screening: Identification of Impaired Vascular Function Using the FMSF Technique. Sensors.

[17] Pabbidi M.R., Kuppusamy M., Didion S.P., Sanapureddy P., Reed J.T., Sontakke S.P. (2018). Sex differences in the vascular function and related mechanisms: Role of 17β-estradiol. Am. J. Physiol. Circ. Physiol..

